# Wastewater surveillance for SARS-CoV-2 during a mass sporting event in the City of Cape Town, Western Cape

**DOI:** 10.3389/fpubh.2024.1462629

**Published:** 2024-12-06

**Authors:** Sizwe Nkambule, Renée Street, Swastika Surujlal-Naicker, Rabia Johnson, Angela Mathee

**Affiliations:** ^1^Environment & Health Research Unit, South African Medical Research Council (SAMRC), Cape Town, South Africa; ^2^Department of Environmental Health, Faculty of Health Sciences, University of Johannesburg, Johannesburg, South Africa; ^3^Scientific Services, Water and Sanitation Department, City of Cape Town Metropolitan Municipality, Cape Town, South Africa; ^4^Biomedical Research and Innovation Platform, South African Medical Research Council (SAMRC), Cape Town, South Africa; ^5^Centre for Cardio-Metabolic Research in Africa, Division of Medical Physiology, Faculty of Medicine and Health Sciences, Stellenbosch University, Stellenbosch, South Africa

**Keywords:** mass gatherings, COVID-19, wastewater, surveillance, public health, sporting event

## Abstract

**Background:**

Wastewater surveillance has become an important public health tool with numerous research studies indicating its potential for monitoring coronavirus disease 2019 (COVID-19) outbreaks. The aim of this study was to apply wastewater surveillance as an indicator for COVID-19 to monitor the impact of a mass sporting event in the City of Cape Town. The study compared the same event over 2 years (2022 and 2023).

**Methods:**

Weekly grab wastewater samples were collected from wastewater treatment plants in the City of Cape Town, and quantitative reverse transcription-polymerase chain reaction used to quantify severe acute respiratory syndrome 2 (SARS-CoV-2) RNA in wastewater.

**Results:**

Our findings show a statistically significant correlation (rho = 0.68, *p* = 0.01) between clinical cases and concentrations of SARS-CoV-2 RNA in wastewater in the 2022 study period. During this specific period, a rise in clinical cases was observed 2 weeks after the event and the peaks in clinical cases coincided with the peaks in SARS-CoV-2 RNA level in wastewater. The study also found a statistically significant positive correlation (*R*^2^ = 0.03, *F* (1,208) = 6.56, *p* = 0.01) between the SARS-CoV-2 RNA in wastewater and the 2022 event of the marathon hosted in the city.

**Conclusion:**

Due to the decrease in clinical testing and the country being a popular destination for mass gatherings such as sporting events, the results from this study indicate the potential of wastewater surveillance providing supplementary information to form part of public health risk evaluations for mass gatherings.

## 1 Introduction

During the coronavirus disease 2019 (COVID-19), wastewater surveillance for severe acute respiratory syndrome coronavirus 2 (SARS-CoV-2) was used as a tool to conduct large scale monitoring in communities (14, 15, 18). It was determined that non-infectious SARS-CoV-2 RNA was detectable in urine and faces and shed in wastewater from both symptomatic and asymptomatic individuals (13). Data obtained from wastewater surveillance was used to infer COVID-19 infections through the correlation of SARS-CoV-2 concentrations in wastewater and reported clinical cases (14, 15, 18). In certain instances, the application of wastewater surveillance for SARS-CoV-2 extended beyond monitoring, and in other instances it was used as an early warning system which assisted in providing targeted public health responses (14, 15, 18). During the 2020 Tokyo Olympics held from July to August, and later the Paralympics which were held from August to September 2021, wastewater-based epidemiology (WBE) was adopted to determine the COVID-19 incidence within the Olympic villages (1). This WBE approach provided information to public health specialists about circulation of variants and informed clinical testing strategies and priorities (1). Mass gatherings, characterized by large crowds and close contact, significantly impact public health, presenting both opportunities and challenges for public health authorities. Such gatherings encompass a wide range of events, including sporting, cultural, religious or political (2), and have been known to place pressure on a host city’s economy, infrastructure, and public health system both in the short and long terms (3). These events can also change population health dynamics of the host city and act as a platform for the transmission of infectious diseases (3, 4). As mass events can attract international participants and spectators, the risk of global transmission of infectious diseases during and after the event may also increase (3, 5). During the COVID-19 pandemic, many countries elected to ban mass gathering events to avert disease transmission (6, 7). These concerns over disease transmission required increased surveillance capacity to respond to outbreaks that can impact human health (8, 4). Wastewater surveillance for SARS-CoV-2 and similar virus can provide supplementary information that reveals disease presence in a community and assist host cities in identifying early signs of disease outbreaks. The surveillance of wastewater can also offer opportunities to conduct community screening of infection levels in areas that may be affected during the hosting of a mass sporting event.

Global responses that have contributed to the management of disease outbreaks following mass gatherings include the World Health Assembly’s call for increased public health measures to reduce health risks in hosting mass events (9). In Africa, mass sporting events such as the 2010 FIFA World Cup in South Africa held during the H1N1 influenza pandemic, and the 2015 Africa Cup of Nations in Equatorial Guinea held during the Ebola virus outbreak demonstrate that global events may be safely held without significant international transmission (6, 9). However, with the COVID-19 pandemic, there were cancelations of international and national sporting events in an attempt to contain the transmission of SARS-CoV-2 (5, 7). These responses may have been spurred by the surge in COVID-19 cases following Biathlon World Championship in Bolzano during March 2020 which was putatively associated with attendees from Germany testing positive for COVID-19 (6). Reflecting on experiences across the world, research suggests that mass gatherings, particularly sporting events, played a part in driving the transmission of SARS-CoV-2 in the early stages of the pandemic (8).

The Two Oceans Marathon (TOM), held annually in the City of Cape Town, is associated with a significant economic boost locally and beyond and is considered Africa’s largest running event. The event attracts local and international athletes with as many as 29,000 participants reported to have been involved in the 2023 edition of the marathon. On 5 April 2022, South Africa lifted its National State of Disaster and introduced transitional measures that were in place from 4 April to 3 May 2022. The transitional measures stated that all persons in an open public space were not required to wear a mask but had to maintain a distance of at least one meter from another person. Persons who were unvaccinated, not fully vaccinated, not in possession of a valid vaccination certificate, not in possession of a valid certificate of a negative COVID-19 test obtained within 72 h before the date of the gathering, were allowed to attend events. However, mass gatherings were limited to 1,000 persons for indoor venues and 2000 persons for outdoor venues. With regard to international travelers, those fully vaccinated were required to produce a valid vaccination certificate, and unvaccinated travelers had to provide a valid certificate of a negative COVID-19 test obtained within 72 h before travel. Due to COVID-19 restrictions, the 2020 and 2021 events of the race did not take place. In 2022, the event was split over 2 days (16 and 17 April) to comply with COVID-19 regulations, with the 2023 marathon taking place on Saturday, 15 April. The TOM offers a unique opportunity to understand disease transmission in one of Africa’s major tourist destinations as a result of hosting a premier annual mass sporting event and the implications for virus transmission. This study seeks to determine the potential of wastewater surveillance for SARS-CoV-2 being used as a tracking tool that can inform COVID-19 surveillance for mass gatherings. As such, wastewater surveillance was undertaken in the City of Cape Town to determine the concentration of SARS-CoV-2 RNA in wastewater and the potential of this wastewater data as supplementary information for COVID-19 surveillance leading up to, during and after the city hosting one of Africa’s biggest running events.

## 2 Method

### 2.1 Wastewater sampling

**Figure 1:**
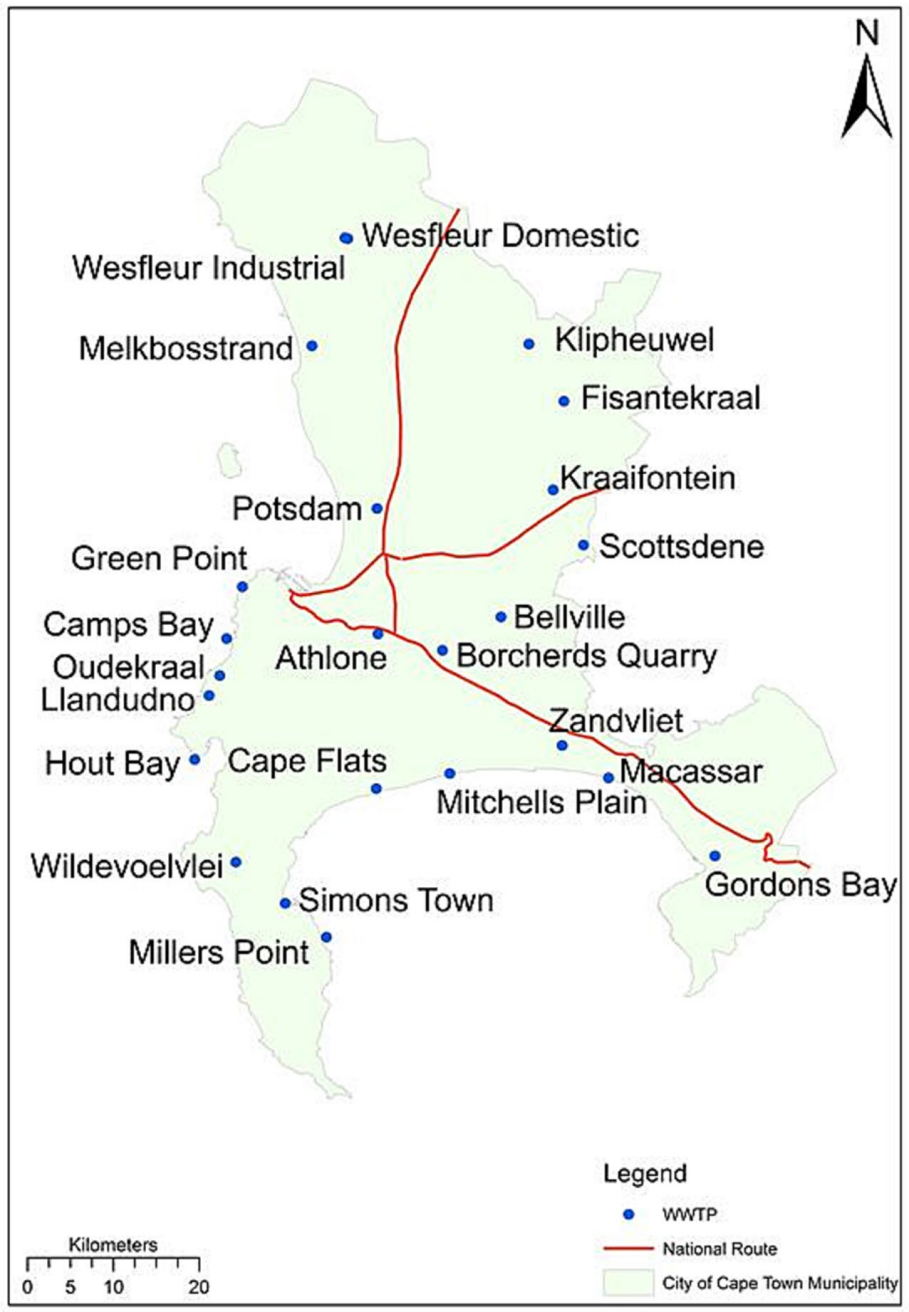
Location of wastewater treatment plants in the City of Cape Metropolitan Municipality, Western Cape, South Africa.

Between 04 April 2022 to 30 May 2022 (year 1), and 03 April 2023 to 29 May 2023 (year 2), 500 mL of grab wastewater samples were collected weekly on Mondays at a similar time each week from the inlet of 24 wastewater treatments plants (WWTPs) in the City of Cape Town (Figure 1). Sampling was conducted in the mornings to capture a period of peak flow of wastewater, along with considerations to ensure samples were delivered by midday to the laboratory on the same day of collection. Measurements on the flow rate of wastewater are not included in this study as this information is not readily available. Wastewater samples were transported to the laboratory on ice for analysis. Samples were collected in the month during, and 1 month after the municipality hosting the Two Oceans marathon. A total of 210 and 115 wastewater samples were collected during the 2022 and 2023 study periods, respectively. Fewer wastewater samples were collected in 2023 due to fewer wastewater treatment plants being monitored.

### 2.2 Sample concentration and RNA extraction

The RNA extraction procedure employed in this study was based on a modified method outlined by (16) and further optimized by Johnson et al. (10). In brief, 100 mL of influent wastewater was centrifuged, and 2–5 mL of the resulting pellet was combined with a 15 mL PowerBead ® Tube containing lysis buffer to deactivate the virus and stabilize the viral RNA (10). Subsequently, the sample underwent homogenization and phase separation, and the total RNA’s quantity and quality were assessed using spectrophotometry with the NanoDrop® ND-1000 instrument (10). To evaluate the efficacy of the extraction method, a clinical SARS-CoV-2 positive nasal swab sample with a known viral load was introduced into a wastewater sample (10). A 10-fold serial dilution was then prepared using the 2019-nCoV-N-positive plasmid control as a standard, provided at 200000 copies/μL (10). The extraction efficiency recovery has been detailed by Johnson et al. (10). In a prior study (11), we evaluated the primary concentration recovery rate for SARS-CoV-2 in a spiked wastewater matrix. To achieve this, a 25 mL SARS-CoV-2-negative passive wastewater sample was spiked with a known concentration of inactivated SARS-CoV-2, either wild-type (E.62) or the 501Y.V2 variant, provided by the Medical Virology Laboratories, Tygerberg. Post-centrifugation, the pellet was resuspended, with viral load calculations based on the resuspension volume and the surface area of the gauze used, as the pellet weight was not recorded. The viral recovery rate was quantified as (copies recovered/copies spiked) × 100. Matrix spike analyses revealed primary concentration recovery rates for attenuated SARS-CoV-2 between 10.59 and 17.41%, consistent with findings from comparable studies.

### 2.3 Quantitative real-time polymerase chain reaction analysis

For the detection of SARS-CoV-2 viral RNA in wastewater samples, the study utilized the quantitative real-time polymerase chain reaction (qRT-PCR) nucleocapsid gene (N1 and N2) primer/probe, which is approved by the Centers for Disease Control and Prevention (CDC) (12). The wastewater samples underwent reverse transcription, amplification, and quantification using the Bio-Rad iTaq Universal Probes One-Step Kit, following the manufacturer’s instructions. To quantify SARS-CoV-2 viral RNA in wastewater, a one-step qRT-PCR reaction was conducted, with duplicate reaction and inclusion of a positive control for each experimental run (10). To minimize the risk of contamination, the RNA extraction and qRT-PCR procedures were carried out in separate laboratories (10). To ensure the sensitivity of the detection method, especially in cases of low viral load, we included a spike RNA control to determine the percentage of viral recovery. This work has previously been reported on by our group in work published by Johnson et al. (10), which utilized an attenuated SARS-CoV-2 strain to estimate recovery efficiency in wastewater. To minimize the risk of false positives due to qRT-PCR sensitivity and to control for contamination, a wastewater control sample from a site with no SARS-CoV-2 was included in all experiments, from extraction through RT-PCR. Additionally, we participated in an interlaboratory comparison as part of the laboratory’s in-house External Quality Assessment (EQA), conducted according to SANAS standards. This included collaboration with two external laboratories from the National Institute for Communicable Diseases (NICD) to ensure the reliability and accuracy of our results.

### 2.4 COVID-19 clinical data

Clinical data on COVID-19 cases was obtained from the News24 COVID-19 Dashboard,^1^ which collated data from the National Department of Health, the National Institute for Communicable Diseases (NICD) and the Western Cape Government. The 7-day moving average is reported as the average number of recorded cases per day over a seven-day period or week.

### 2.5 Statistical analysis

Summary statistics were used to describe the SARS-CoV-2 RNA copies per milliliter of wastewater, with RNA concentrations below the limit of detection (LOD) (700 copies/mL) replaced with values half of the LOD. For analysis and plotting of data, the RNA values were log transformed. Due to the lack of readily available flow rate across all wastewater treatment plants in this study area, RNA concentrations have been presented without normalization. To determine the correlation between the N1 and N2 primers, Spearman’s rank correlation was performed at a significance level of 0.05. The N1 and N2 values were combined, and the average value was used as a single measurement.

This study took place over a nine-week period for each edition of the marathon, analyzing data for the month of the event as well as the following month. Wilcoxon signed rank test was performed to conduct a paired difference test of SARS-CoV-2 RNA viral load in wastewater during the 2022 and 2023 study periods of the Two Oceans marathon and assess whether the mean ranks differ. Using Time Series regression, the impact of the Two Oceans marathon in 2022 and 2023 was assessed to determine whether there was a statistically significant change in SARS-CoV-2 RNA signal in wastewater that is associated with the occurrence of the marathon. The time series regression used a distributed lag model. The *R*^2^ denotes how well the model captures variations in data, the F-statistic and its *p*-value test whether the overall regression model is statistically significant. The weekly median SARS-CoV-2 RNA signal in wastewater was only used to compare with the reported 7-day moving average of COVID-19 cases in the City of Cape Town for the 2022 study period only. Comparisons could not be made for the 2023 study period as the Western Cape Department of Health had issued a notice that as of 6 April 2023, testing rates were extremely low across the country and could no longer provide an accurate proxy for COVID-19 cases.

## 3 Results

Spearman’s correlation indicated a strong and positive relationship between the 2022 N1 and N2 values (rho = 0.86, *p* = 0.01) and the 2023 N1 and N2 values (rho = 0.68, *p* = 0.01). During the 2022 study period, 92% (194/210) of wastewater samples tested positive for SARS-CoV-2 RNA with the highest viral load equalling 30,117 copies/mL (wastewater samples collected on 9 May 2022) and a median of 3,230 copies/mL. In the 2023 study period, 70% (81/115) of wastewater samples tested positive for SARS-CoV-2 RNA with the highest viral load equalling 6,761 copies/mL (24 April 2023) and a median of 350 copies/mL. The difference in the number of wastewater samples testing positive for SARS-CoV-2 RNA between the 2022 and 2023 can also be linked to the difference in correlation coefficients. Because there were fewer positive samples in 2023, there were fewer data points to make comparisons in N1 and N2, thus reducing statistical power and influencing the correlation. Table 1 shows the median paired difference of SARS-CoV-2 RNA signal in wastewater during the study week period of the 2022 and 2023 Two Oceans marathon events. This was done to determine the difference in viral loads given the differences in COVID-19 regulations and prevalence in the country for each of the years the event was held. The results indicate that the median SARS-CoV-2 RNA signal was higher during the 2022 Two Oceans marathon in comparison to the 2023 event of the race, except for study week 1. Moreover, there was a statistically significant difference in the median SARS-CoV-2 RNA signal except for study weeks 1 and 3 in the 2022 and 2023 events of the race. The median differences could not be determined for week 5 as there were no wastewater data available during this time period in 2023, due to the day of sampling being a public holiday. Missing data may present challenges such as reducing statistical power and representativeness, or cause bias. However, in this study, the missing data for 2023 only limits comparison of mean differences for the corresponding week in 2022. The missing 2023-week 5 data thus had no impact on the whole dataset as the paired differences in SARS-CoV-2 RNA were done to compare corresponding weeks.

**Table 1:** Paired difference of median SARS-CoV-2 RNA signal in wastewater during the study week of the 2022 and 2023 Two Oceans Marathon.

Study week	Dates	Paired difference	*p* value	2022 median RNA concentration (copies/ml)	2023 median RNA concentration (copies/ml)
1	04 April 2022; 03 April 2023	−0.67	0.50	1,433	2000
2	11 April 2022; 10 April 2023[week of event]	2.666	0.01	2,459	275
3	18 April 2022;17 April 2023	0.622	0.57	1,687	976
4	25 April 2022; 24 April 2023	3.357	0.01	2,347	886
5	02 May 2022; 01 May 2023	Not determined	Not determined	7,093	Not determined
6	09 May 2022; 08 May 2023	3.599	0.01	2,941	416
7	16 May 2022; 15 May 2023	2.666	0.01	3,379	346
8	23 May 2022; 22 May 2023	2.457	0.02	8,798	416
9	30 May 2022; 29 May 2023	2.201	0.03	3,969	231

**Figure 2:**
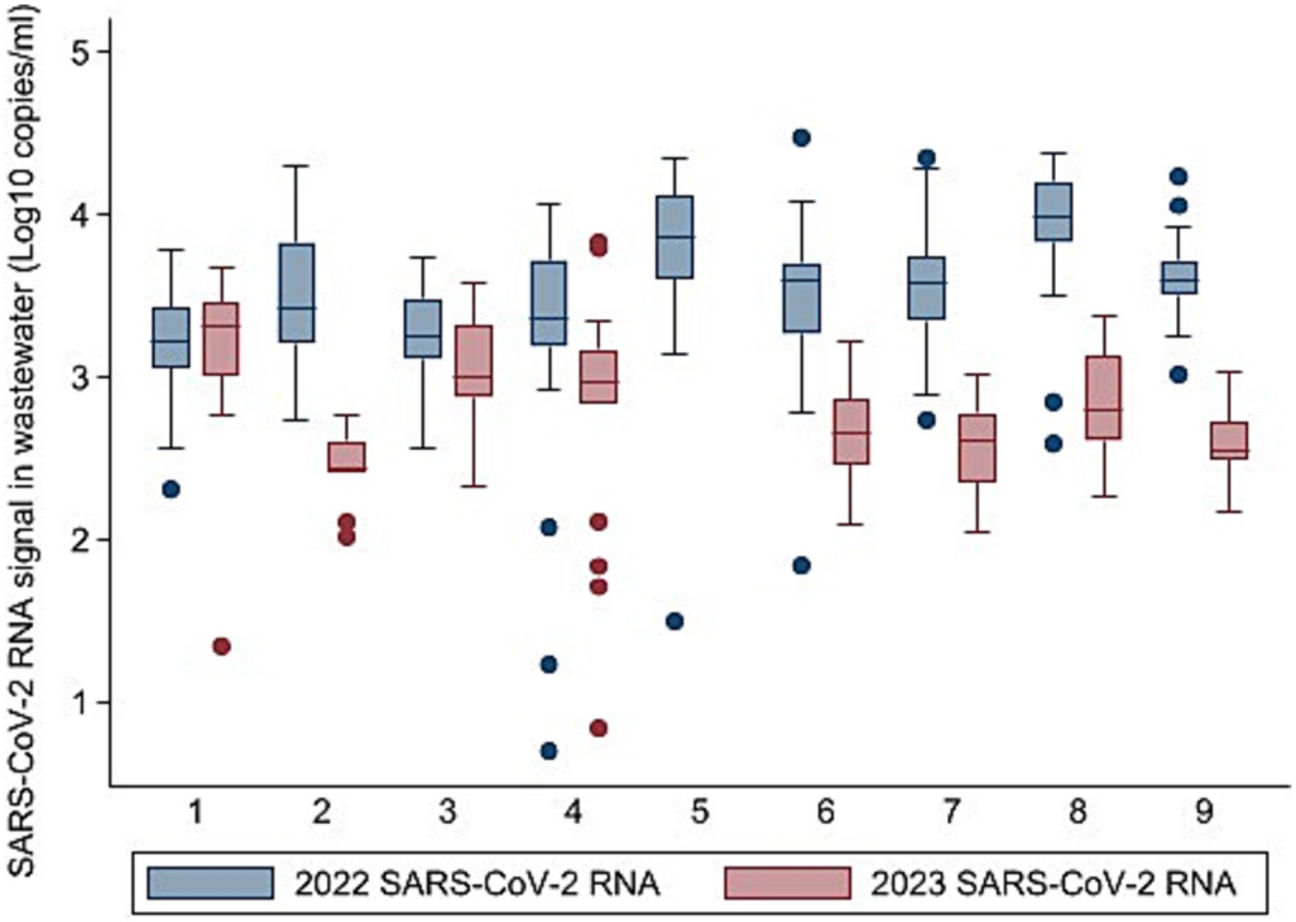
Trends in SARS-CoV-2 RNA by wastewater treatment plant (WWTP) during the nine-week period of the 2022 and 2023 edition of the Two Oceans Marathon.

Figure 2 shows the trends in SARS-CoV-2 RNA during the nine-week period for the 2022 and 2023 events of the marathon. The orange dotted line indicates the week subsequent to the marathon. Time Series regression was also performed to assess the impact of the TOM on SARS-CoV-2 RNA signal in wastewater. There was a statistically significant positive correlation (*R*^2^ = 0.03, *F* (1,208) = 6.56, *p* = 0.01) between the SARS-CoV-2 RNA in wastewater and the occurrence of the 2022 marathon. This indicates that the increased SARS-CoV-2 RNA signal in wastewater is associated with the city hosting the mass event. During the 2023 event, the study also found a statistically significant correlation (*R*^2^ = 0.05, *F* (1,113) = 6.04, *p* = 0.02) between the SARS-CoV-2 RNA signal in wastewater and the mass event; however, the regression coefficient indicates that the correlation was negative. This negative correlation suggests that during and after the event in the 2023 study period, the concentration of SARS-CoV-2 RNA in wastewater decreased, rather than increased as observed in the previous year. When the TOM took place in 2023, it had approximately been 1 year since the COVID-19 regulations had been lifted, vaccination programs had been widespread in country, and testing rates in the city and province had been decreasing. As such, it is likely that when the event took place in 2023, despite there being an influx of visitors, the population consisted of fewer people shedding the virus in wastewater, which in turn can explain why the mass event did not lead to an increase in SARS-CoV-2 RNA in wastewater.

**Figure 3:**
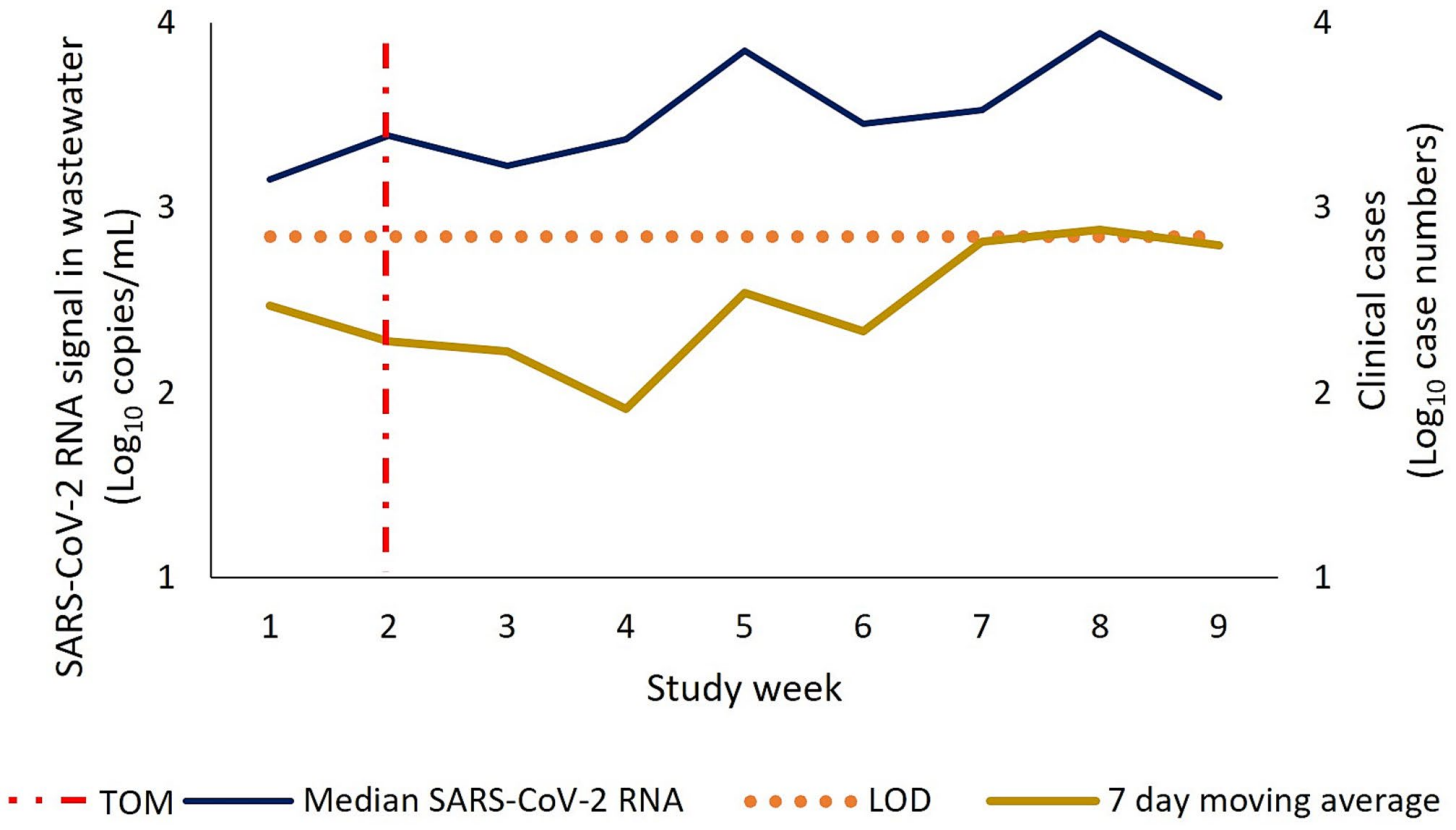
Trends in median SARS-CoV-2 RNA in wastewater and clinical cases reported in the City of Cape Town during the 2022 study period.

This study also compared trends between the clinical cases in the City of Cape Town and the SARS-CoV-2 RNA detected in wastewater (Figure 3). The trends could not be compared for 2023 as clinical cases were no longer reported by the city. There was a moderately strong and significant correlation between the clinical cases and SARS-CoV-2 RNA signal detected in wastewater during the 2022 study period (rho = 0.68, *p* = 0.01). The highest median RNA signal of 8,798 copies/mL was observed in study week 8 (23 May 2022), with the highest number of clinical cases also reported in the same week. Between study week 3 (18 April 2022) which represented the first day of sampling post the marathon, and study week 5 (02 May 2022) there was a two-fold increase in clinical cases from 168 to 347 in this two-week period. A similar trend was observed in the wastewater data in this two-week period with the median RNA signal increasing from 1,687 copies/mL in study week 3 to 7,093 copies/mL in study week 5. Furthermore, between study weeks 6–8, the trend in RNA signal in wastewater increased each consecutive week, coinciding with an increased trend in clinical cases over that same time period.

## 4 Discussion

Comparisons between the viral load in wastewater indicated significantly higher amplitude in the presence of SARS-CoV-2 RNA in the 2022 study week. This is to be expected since the country was undergoing a transition in COVID-19 regulations which were associated with less restrictions for mass gathering events as of 04 April 2022, which was 2 weeks prior to the commencement of the Two Oceans marathon on 17 April 2022. In addition, the circulating strain and the combination of strain virulence and mass gatherings could have contributed to the spread and increase in the viral load in wastewater. In April and May 2022, the highly transmissible Omicron variant was the predominant variant of concern circulating in South Africa which accounts for higher concentrations of SARS-CoV-2 RNA shed into wastewater. Furthermore, the levels of SARS-CoV-2 RNA in wastewater is understandably low during the 2023 study period, as at this point the vaccination rollout in the country was widespread, a significant portion of the population had become immune to the virus either through vaccination or previous infection, and COVID-19 cases were low. To the best of the authors knowledge, there were no public health interventions implemented after the event in both years, that would have otherwise influenced clinical cases.

This study found a positive and statistically significant correlation between the clinical cases reported in the City of Cape Town and the SARS-CoV-2 RNA signal in wastewater in the month during and after the Two Oceans marathon was hosted by the municipality. This implies that mass gatherings may have influenced the spread of COVID-19, inferring that such events could act as superspreader events and as such its important to monitor their impact on public health. However, it is important to note that when making associations from wastewater surveillance, the SARS-CoV-2 RNA concentrations are indicative of how much virus is shed into wastewater, and not reflective of the number of individuals shedding the virus. The wastewater data should not be interpreted in isolation, but rather used in conjunction with other health data. As, findings from this study suggests, wastewater surveillance can be used as supplementary information on trends of COVID-19 cases that can inform public health authorities determine the health impacts of hosting mass gathering events. These findings are similar to those made by Kitajima et al. (1) that used wastewater surveillance to track COVID-19 in the Tokyo 2020 Olympic and Paralympic Village. Of particular interest, the current study was able to assess trends from samples collected at wastewater treatment plants which are part of centralized sewer systems, unlike the Kitajima et al. (1) study where samples were collected from manholes around the village. This indicates the ability of using sampling locations downstream in the sewer system to track SARS-CoV-2 RNA in wastewater as part of surveillance for mass gatherings.

Prior to the commencement of the sporting event in April 2022, clinical cases in the city had been decreasing. Two-weeks post the city hosting the 2022 event, there was a noticeable increase in both the clinical case data and RNA signal in the wastewater, with both data reaching their peaks in similar weeks. Similarly, a study by Alahmari et al. (6) in Pesaro, Italy, reported on an increase in cases 15 days post the city hosting a sport and cultural events which were identified as driving the spread of COVID-19. During the 2023 study week, the Western Cape government had ceased to provide an update on clinical cases as of 06 April 2023 on their COVID-19 dashboard, citing extremely low testing rates which could no longer act as an accurate proxy of COVID-19 cases. This further gives motivation for the use of wastewater surveillance, especially in the absence of accurate testing information.

As South Africa continues to host mass gatherings such as sporting events that drawn in international and local travelers, it is important to consider potential health impacts in the host city, as these can become superspreading events that potentially transmit infections within communities and across borders. Wastewater surveillance can be used as a tool in broader public health risk assessments for mass gathering events during outbreaks. The WHO has made recommendations for managing public health risks linked to mass gatherings and devised a risk assessment tool on how to evaluate event risks in light of COVID-19. The assessment tool includes a section on the evaluation of COVID-19 transmission patterns in the country/city the event is hosted, disease surveillance and detection, as well as detection and monitoring event-related COVID-19 (6, 17). Given the similarities in trends between the clinical cases and wastewater data, this study thus recommends wastewater surveillance data to form part of public health strategies that seek to monitor how hosting a mass sporting event may affect the presence and amplitude of virus that can be spread during such events.

A limitation of the study is that grab wastewater samples were collected, reflective of one-time point in the week. However, the routine nature of sampling did allow for trends in SARS-CoV-2 concentration in wastewater to be observed. The study was unable to make comparisons between the wastewater data and clinical cases in 2023 as the Western Cape government had ceased providing updates on clinical case data. Despite this, the correlation between the datasets in 2022 during and after the event are indicative of the potential in using wastewater surveillance to infer on COVID-19 infections for cities hosting mass gathering events.

## 5 Conclusion

This study reports on the detection of SARS-CoV-2 RNA in wastewater samples collected across the City of Cape Town and demonstrates the utility of monitoring wastewater during and after mass gathering sporting events in an African setting. This was the first study to demonstrate this utility using sampling locations downstream the sewer system rather than sampling from more defined communities. The similar trends observed between the clinical cases and wastewater data indicates the importance of this surveillance approach as part of public health risk assessments for mass gathering events, in the absence of accurate testing information. The data obtained through wastewater surveillance can provide beneficial information for stakeholders which can assist in developing targeted clinical testing in hotspot areas, and other health advisories. From a policy perspective, wastewater surveillance can be included in guidelines for host cities, offering a novel approach to monitoring the transmission of SARS-CoV-2 and similar viruses during and post mass gatherings. Due to wastewater samples being collected weekly, this study recommends further research where there is increased frequency of sampling within a week to determine any fluctuations in the days leading up to the city hosting the sporting event.

## Data Availability

The original contributions presented in the study are included in the article/supplementary material, further inquiries can be directed to the corresponding author.

## Footnotes

^1^https://covid-19dashboard.news24.com/
